# VIPER: Visualization Pipeline for RNA-seq, a Snakemake workflow for efficient and complete RNA-seq analysis

**DOI:** 10.1186/s12859-018-2139-9

**Published:** 2018-04-12

**Authors:** MacIntosh Cornwell, Mahesh Vangala, Len Taing, Zachary Herbert, Johannes Köster, Bo Li, Hanfei Sun, Taiwen Li, Jian Zhang, Xintao Qiu, Matthew Pun, Rinath Jeselsohn, Myles Brown, X. Shirley Liu, Henry W. Long

**Affiliations:** 1 Department of Medical Oncology, Dana-Farber Cancer Institute, Boston, MA 02215 USA; 2 Center for Functional Cancer Epigenetics, Dana-Farber Cancer Institute, Boston, MA 02215 USA; 3 Department of Biostatistics and Computational Biology, Dana-Farber Cancer Institute and Harvard School of Public Health, Boston, MA 02215 USA; 4 Institute of Human Genetics, University of Duisburg-Essen, Essen, Germany; 5 University of Massachusetts Medical School, Worcester, MA 01655 USA; 6 Molecular Biology Core Facilities, Dana-Farber Cancer Institute, Boston, MA 02215 USA; 7 Department of Bioinformatics, School of Life Sciences, Tongji University, Shanghai, 200092 China; 8 State Key Laboratory of Oral Diseases, West China Hospital of Stomatology, Sichuan University, Chengdu, China; 9 Beijing Institute of Basic Medical Sciences, Beijing, China

**Keywords:** RNA-seq, Analysis, Pipeline, Snakemake, Gene fusion, Immunological infiltrate

## Abstract

**Background:**

RNA sequencing has become a ubiquitous technology used throughout life sciences as an effective method of measuring RNA abundance quantitatively in tissues and cells. The increase in use of RNA-seq technology has led to the continuous development of new tools for every step of analysis from alignment to downstream pathway analysis. However, effectively using these analysis tools in a scalable and reproducible way can be challenging, especially for non-experts.

**Results:**

Using the workflow management system Snakemake we have developed a user friendly, fast, efficient, and comprehensive pipeline for RNA-seq analysis. VIPER (Visualization Pipeline for RNA-seq analysis) is an analysis workflow that combines some of the most popular tools to take RNA-seq analysis from raw sequencing data, through alignment and quality control, into downstream differential expression and pathway analysis. VIPER has been created in a modular fashion to allow for the rapid incorporation of new tools to expand the capabilities. This capacity has already been exploited to include very recently developed tools that explore immune infiltrate and T-cell CDR (Complementarity-Determining Regions) reconstruction abilities. The pipeline has been conveniently packaged such that minimal computational skills are required to download and install the dozens of software packages that VIPER uses.

**Conclusions:**

VIPER is a comprehensive solution that performs most standard RNA-seq analyses quickly and effectively with a built-in capacity for customization and expansion.

**Electronic supplementary material:**

The online version of this article (10.1186/s12859-018-2139-9) contains supplementary material, which is available to authorized users.

## Background

Transcriptome sequencing is now a commonplace technique employed in many disparate scientific settings [1–4]. The decrease of cost and rapid development of simple kits for this technology has enabled researchers to use transcriptome sequencing (RNA-seq) as a common and essential method for probing the underlying transcriptional behavior of cells and tissues.

Current next-generation sequencing methods yield fastq files that contain the sequencing reads captured from the sample. These reads are typically aligned to a specific reference genome. In RNA-seq, the reads after alignment are quantified on a per gene or per transcript basis to discern information regarding the level of gene expression in a population of cells. Additional analyses may include technical quality control of the sequencing libraries and clustering analysis for experimental quality control. Often, analysis is done to compare samples of two conditions against each other, and determine the statistically significant differences in the level of transcripts per gene. Further analysis can investigate the pathways associated with these differentially expressed genes, perform various read metrics to assess the variability of the data, and identify single nucleotide changes or deletions that occur throughout the coding regions or the genome.

In this contribution we address the problem of creating robust, easily adaptable software for the quality control and analysis of RNA-seq data. This is a difficult problem because the field is moving very rapidly with new and improved algorithms for key tasks being published frequently. Also novel applications of RNA-seq are constantly being enabled by new analytic approaches. For example innovations in analysis now permit tools to be developed that aid in the discovery of fusion genes [5–7], the identification of viral transcripts [8, 9] and the analysis of immunological infiltrate in samples [10, 11], which enable a deeper understanding of the biological system being studied.

Although some aspects of RNA-seq analysis are becoming more standard, the number of bioinformatics tools to choose from can be overwhelming. Furthermore, installing the desired tools and all requisite dependencies is often non-trivial. Lastly, maintaining such a system while allowing for the rapid modification to accommodate new analyses is a challenging task.

Other groups have addressed these issues and a common solution is to piece together several tools to create a single pipeline, through which one can then process their data while minimizing hands on time and optimizing the choice of each underlying algorithm. Numerous pipelines have been reported in the literature [12–14] but there is still a strong need for new pipelines that are easy to modify to allow new analysis methods to be added onto the existing ones and can be used by people of all levels of computational experience.

The system presented here, VIPER (Visualization Pipeline for RNA sequencing analysis), uses a modern computational workflow management system, Snakemake [15], to combine many of the most useful tools currently employed in RNA-seq analysis into a single, fast, easy to use pipeline, that includes alignment steps, quality control, differential gene expression and pathway analyses. In addition, VIPER includes a variety of optional steps for variant analysis, fusion gene detection, viral DNA detection and evaluation of potential immune cell infiltrates. VIPER was built with three guiding principles. (1) Highly modular pipeline exploiting the Snakemake framework that allows for rapid integration of new approaches or replacement of existing algorithms. (2) Visual output for rapid “at a glance” insight with detailed results from each analysis step available in a well-defined folder hierarchy. (3) Can be run using simple command line entries by the inexperienced, while maintaining the ability to be fully customizable by users who have more experience with writing and deploying computational biology tools. Using these principles we have created a flexible analysis pipeline that carries out many standard tasks, adds several very recently developed algorithms for immunological analysis and can be rapidly extended when new capabilities are required.

## Implementation

**Fig. 1:**
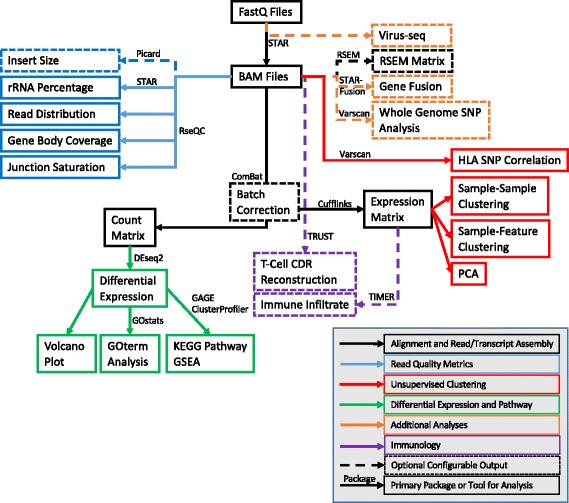
Overview of the full workflow performed by VIPER (Visualization Pipeline for RNAseq analysis). The different segments of the pipeline are broken down by color. The core of the pipeline is the read alignment performed by STAR that outputs alignment (bam) files. Gene expression is quantitated with Cufflinks for unsupervised analysis (clustering and PCA). STAR also generates a count matrix used for supervised analysis (differential expression with DESeq2). When a publically available analysis tool is used for a particular step, the name of the tool is identified above the arrow leading to the resulting output (boxed). When there is no tool indicated next to an arrow, the analysis step was performed with custom R code. Conditional/optional analyses are denoted with a hashed arrow and outlining box and represent the most distinguishing functionality for VIPER

The analysis steps of VIPER are expressed in terms of “rules” connecting input files to output files as part of the overall workflow (Fig. 1). Upon execution, Snakemake *infers* the combination of rules necessary to achieve a “target” or specific output, in our case the final report. The necessary steps are run in an optimized manner depending on the computational environment [15]. This inference allows for rules to be swapped out transparently if the inputs and outputs remain the same, e.g. changing an alignment algorithm. VIPER runs from a single configuration file (referred to as the *config* file), where the user lists their fastq files and certain parameters pertaining to the analysis using the human readable yaml format (Additional file 1). VIPER uses a single csv file, containing metadata about the samples and the differential analyses to be performed that can be generated with Excel (referred to as the *metasheet*) (Additional file 2). Running the pipeline requires a single command, and the output is all stored into a single folder, containing easy to navigate subfolders that host the generated analyses (Additional file 3: Figure S2). A significant and unique advantage to VIPER is that its underlying framework enables easy and efficient rerunning of analyses. Unless the relevant input files have been changed, upstream steps of the pipeline will not be re-executed. The user can easily re-execute steps if errors have occurred or the data needs to be subsetted or parameters adjusted.

The overall VIPER workflow (Additional file 4: Figure S1) is comprised of spliced alignment of raw reads to a reference genome to generate raw and normalized counts; a variety of quality checks of mapped reads; Clustering of samples based on gene expression levels; differential expression (DE) testing of genes across samples and Pathway analysis of differentially expressed genes. In addition to these core functionalities, VIPER currently contains several optional modules: (1) RSEM quantification, (2) SNV (single nucleotide variant) identification, (3) Gene fusion detection, (4) Batch effect correction, (5) Virus analysis and (6) analysis of immune cell infiltrate. Below we briefly review which algorithms VIPER uses at each stage.

## Results

To illustrate the utility of VIPER we applied it to a set of patient derived xenografts from bone marrow and blood specimens from patients with leukemia and lymphomas [16]. This publically available paired-end RNA-seq dataset contains eight B-cell acute lymphoblastic leukemia (B-ALL), three T-cell ALL (T-ALL), and three blastic plasmacytoid dendritic cell neoplasm (BPDCN) samples. These are the official World Health Organization (WHO) categories defining these malignancies; additional metadata is in Additional file 2.

### Read alignment, counting and transcript assembly

VIPER uses STAR [17] as the default aligner. The STAR aligner is known for its superior speed that integrates very well with Snakemake’s underlying ability to allocate resources and execute multithreaded processes. The read alignments from STAR are stored in a binary alignment/mapping (BAM) file. Cufflinks [18] is used to assemble transcripts and obtain normalized read counts per gene and isoform in terms of FPKM values. For the user’s convenience in visualizing data in a genome browser, VIPER also converts all the BAM files into BigWig format using Bedtools [19]. In addition, if the input data are paired end, VIPER’s Gene Fusion module, which uses STAR-Fusion [20, 21], will be triggered automatically, and will output fusion genes discovered during alignment. Several custom scripts are added into VIPER to graphically represent the alignment and fusion genes information. In all, the resulting gene and transcript counts are returned as a raw count file from STAR, a normalized gene count from Cufflinks, and optionally, an RSEM formatted file if the user desires this output for further analysis.

### Read quality metrics

**Fig. 2:**
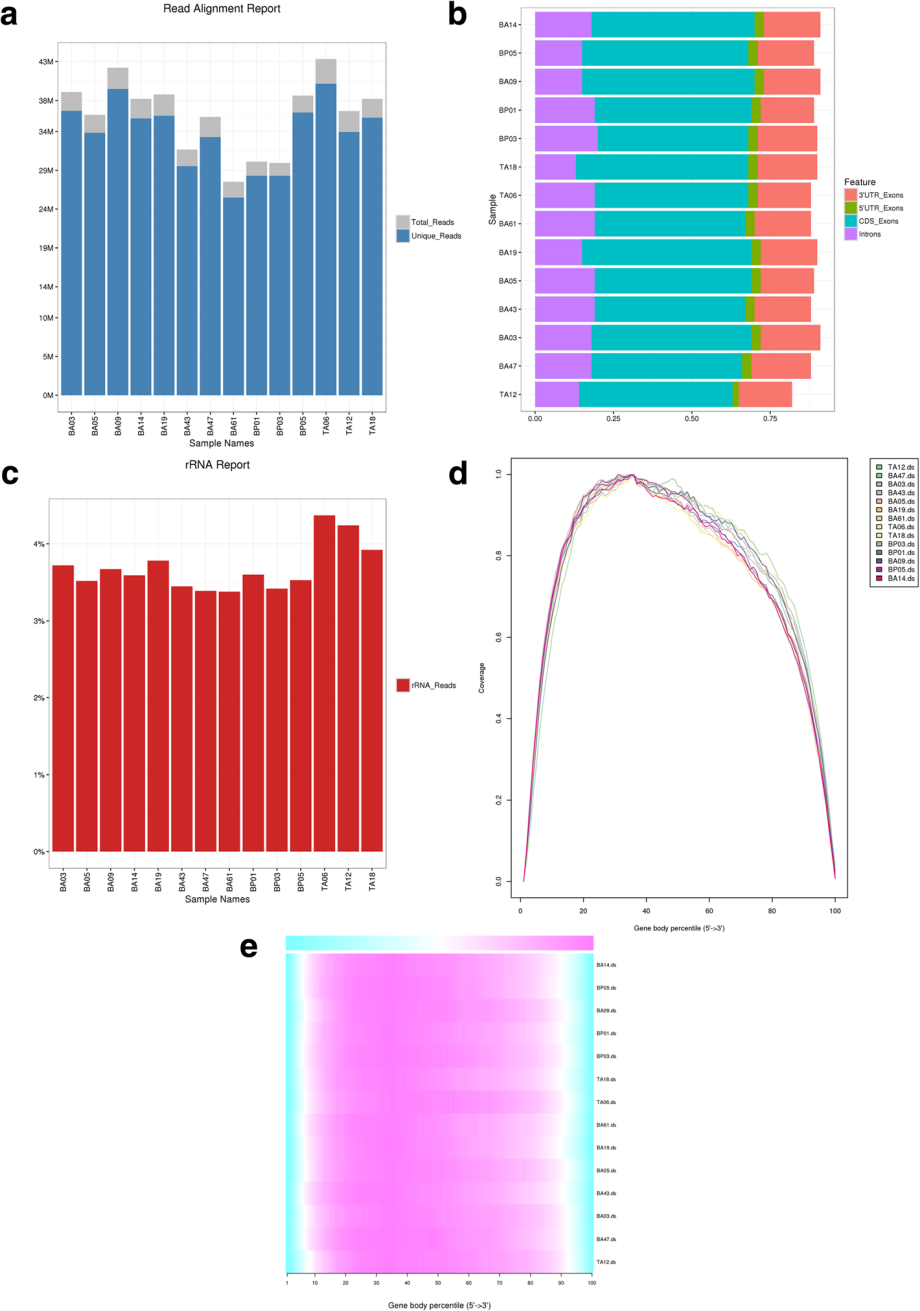
**a** Read Alignment Report denoting the number of mapped and uniquely mapped reads per sample. **b** Read Distribution Report illustrating the percentage of reads that fall into specific genomic regions. **c** rRNA Read Alignment Report demonstrating the percentage of each sample that were considered rRNA reads. Gene Body Coverage of the samples illustrated as (**d**) curves and as (**e**) bars in a heatmap

The alignment output is further investigated to assess the quality of raw reads (Fig. 2). In order to expedite the read quality assessment without compromising on statistical meaningfulness of variability in raw reads, we integrated down sampling of raw reads (to 1 million reads) using the Picard [22] DownsampleSam tool. We have integrated RSeQC [23] to capture read quality metrics such as read distribution, gene body coverage and rRNA contamination. Of note, the RSeQC package was heavily modified to make it amenable to parallel processing in grid/multi core environment. Specifically, the tools that make up RseQC were parsed out into individual rules to allow for 1) parallel processing that significantly increases analysis speeds and 2) and adding scripts that process the RSeQC output to be as readable and user friendly as possible. The xenograft data show uniformly high quality read metrics as expected from a published dataset. There are similar numbers of reads for each sample with high mapping rates (Fig. 1a) representing reads that are mostly in exons and UTRs (Fig. 1b). The coverage of these reads over gene bodies is quite uniform (Fig. 1d, e) and ribosomal reads are all comparable and at a relatively low level (Fig. 1c).

### Unsupervised clustering of samples

**Fig. 3:**
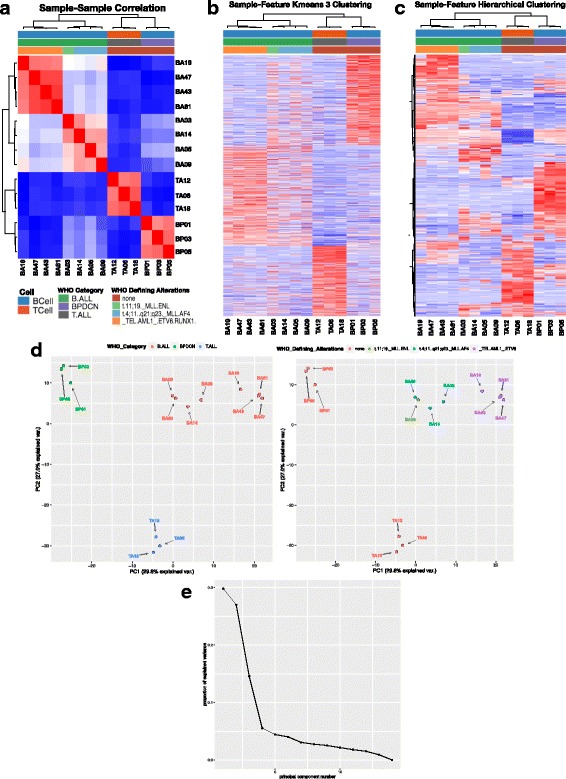
**a** Sample-Sample Clustering Map depicting samples on both axes with the color representative of the correlation between samples. Metadata columns (provided by the user) are annotated along the top. **b** Sample-Feature (Gene) Hierarchical Clustering Map with samples along the x-axis and genes along the y-axis. Metadata columns (provided by the user) are annotated along the top. **c** Sample-Feature heatmaps can also be plotted using k-means clustering, with the number of clusters being configured in the input file. **d** Principal Component Analysis (PCA) plots, with one being output per metasheet column with the coloring corresponding to the metadata within the column. **e** Scree plot depicting the amount of variance captured within each principal component

After alignment is completed and quality control measurements are taken, VIPER uses the count matrix from STAR and the expression matrix from Cufflinks to perform downstream analysis. This begins with unsupervised clustering to look for patterns within the data. VIPER has configurable parameters for filtering genes, such that it will only use genes that pass a configured FPKM threshold and are seen in a user determined number of samples (default is two). VIPER takes the filtered expression data and generates three initial figures for the overview of the sample data (Fig. 3).

First, VIPER will output a Sample-Sample Correlation heatmap, determining the correlation between all of the samples on a pairwise basis. Metadata (provided by the user) are used to annotate samples along the top. In Fig. 3a the xenograft data shows clear clustering by category (B-ALL, T-ALL, BPDCN) based on the sample dendrogram at the top of the figure as well as the differences in the degree of correlation observed between groups vs. in group seen in the heatmap. Secondly, VIPER will output a Sample-Feature heatmap which will show the clustering of samples based on correlation on the horizontal axis and a user configured number of features, or genes, on the vertical axis that can be ordered by hierarchical or k-means clustering (where k is simply specified in the configuration file as one or multiple values). In Fig. 3b and c one sees the same sample clustering along the top as in Fig. 3a and clear groups of genes that are upregulated in the different sample groups in the heatmap. Finally, VIPER will output a Principal Component Analysis (PCA) plot depicting how samples cluster across the first two principal axes (those with the largest variance) and, if metadata is provided for these samples, they will be color coded by the provided annotations. The xenograft samples are clearly clustered based on the different WHO categories colored in the first PCA plot (Fig. 3d). In the second PCA plot the coloring allows one to see a clear separation between the B-ALL samples based on WHO Defining Alterations, namely those with a MLL gene rearrangement and those with an ETV6 fusion. These unsupervised plots provide a preliminary view of the data to determine if any overarching patterns exist between the samples, whether any outliers exist and, using the Sample-Feature map, which genes may be forcing the clustering of samples [24].

### Differential expression and pathway analysis

**Fig. 4:**
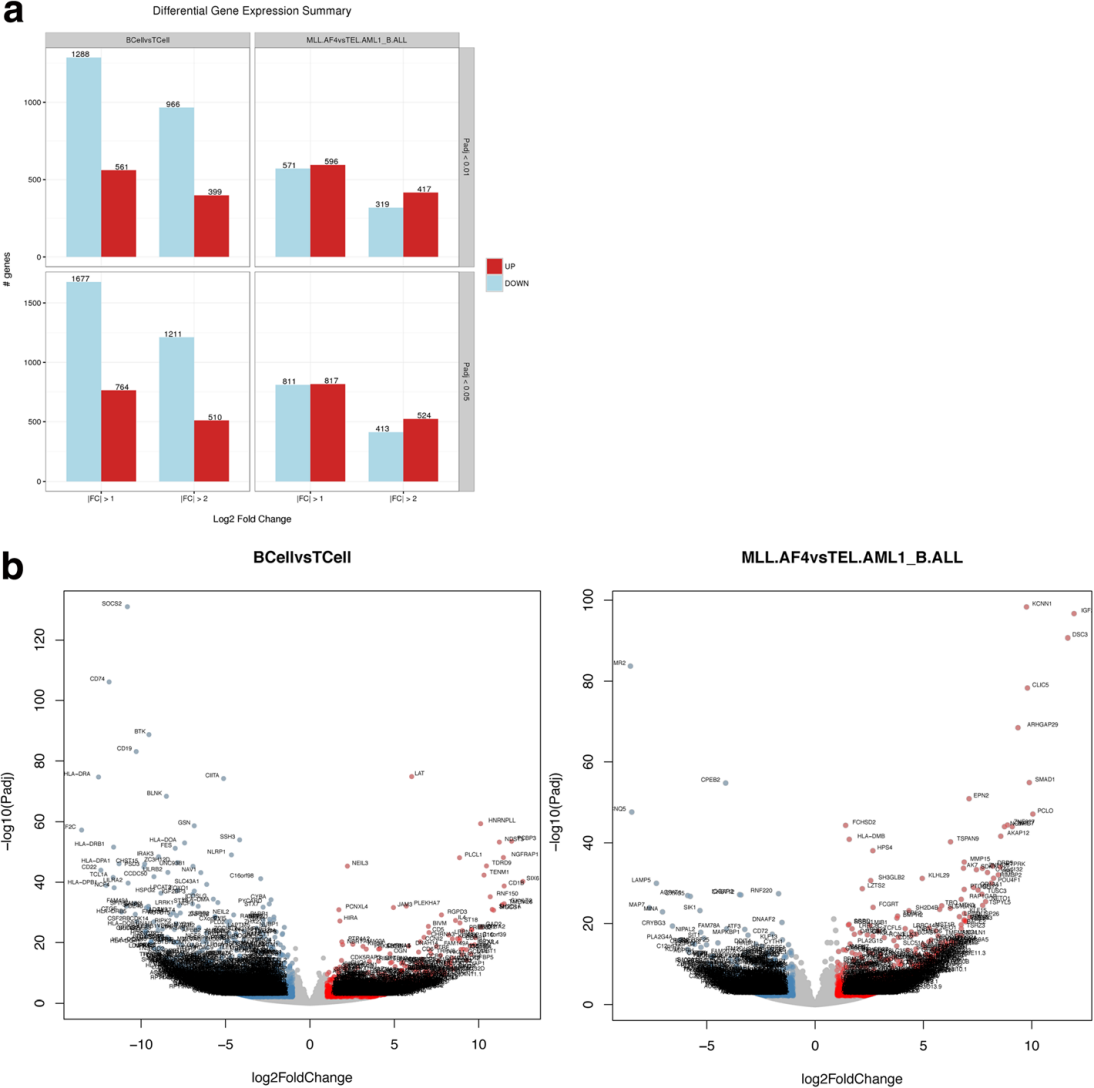
**a** Differential Gene Expression Summary plot summarizing the number of up and down regulated genes per comparison, broken down by various P_adj_ (adjusted *p*-value) and Log2 Fold Change cutoffs. **b** Volcano Plot visually representing the each of the differential expressions in the VIPER run, labeled points have a P_adj_ < 0.01, and an absolute Log2 Fold Change > 1

The first step of the downstream analysis is to determine the differential expression of genes within the user-defined comparisons. Differential expression analysis can be done using several tools that are currently available, with differing models and advantages [25]. There are a number of opinions on which differential expression tools are best [4, 25–28] and VIPER’s modular framework could theoretically enable a user to build in whichever differential expression method that is desired. Based upon literature review and also their wide spread use, we opted for DEseq2 [29] and Limma [30]. Outputting both analyses enables users to confirm results across two leading methodologies, but for the purpose of being as conservative and accurate as possible [26], we have elected to use DEseq2 results for further downstream expression analysis. For each comparison the number of differentially expressed genes for two Padj cutoffs and two Fold Change cutoffs is displayed in a simple bar chart showing both up and down-regulated genes (Fig. 4a); a volcano plot is also shown (Fig. 4b). For the xenograft samples we see a very large number of genes differentiating the B-cell malignancies from the T-cell malignancies as would be expected for such distinct lineages. There are also a significant number of differentially expressed genes between the subtypes of B-ALL; since these are defined by distinct rearrangements of transcription factors this is also expected.

**Fig. 5:**
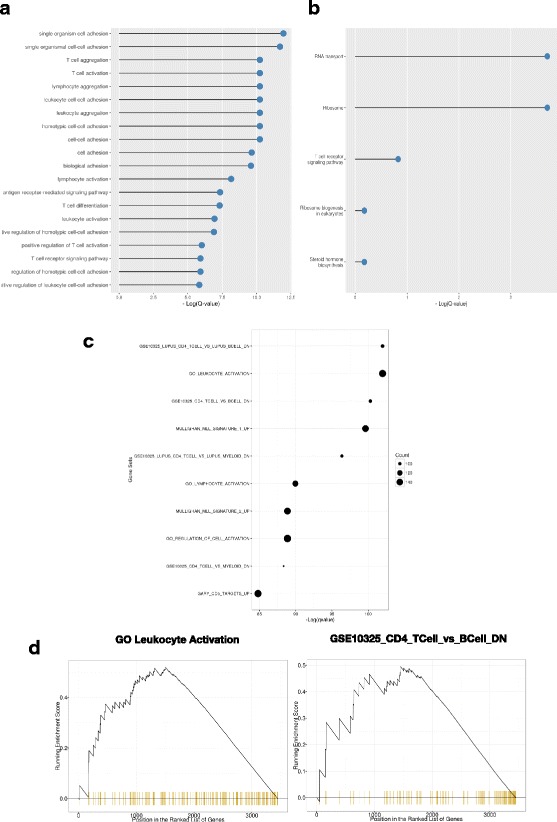
Summary plot depicting the results of analyzing the differentially increased genes for enrichment (**a**) in GO terms (**b**) KEGG pathways and (**c**) MSigDB gene sets. There are corresponding plots (not shown) showing top differentially decreased pathways. **d** A plot showing the running enrichment score of the indicated gene sets within the ranked list of differentially expressed genes

The DEseq2 table from each comparison is subsequently used by a number of tools to perform the gene set and pathway analysis associated with this differential expression (Fig. 5). Gene Ontology (GO) term analysis is also a useful tool to categorize differentially expressed genes. Using GOstats [31] we take in all of the genes that meet a user defined false discovery rate (set in the config file), and extract all of the GO terms associated with this gene set.

KEGG pathway analysis is another fundamental tool for exploring how differentially expressed genes are related on a systematic basis. Using the GAGE [32] package, VIPER takes the entire set of differentially expressed genes, and searches for KEGG pathways significantly associated with the expression differences (Fig. 5b). Using the Pathview package [33], VIPER will also output detailed figures depicting the individual genes within their pathway and their respective expression changes. Finally, Gene Set Enrichment Analysis (GSEA) is also performed. This outputs the top scoring gene sets (Fig. 5c) against MSigDB using the tool ClusterProfiler [34]. We note that this can be used to test for enrichment against user-defined signatures by expanding the text file holding the reference signatures.

As per the VIPER guiding principles, each of these analyses is accompanied by a useful figure that depicts the key aspect of the analysis and the associated table of the underlying data, which can be useful for further investigation. All of this is output into an easy to navigate folder (Additional file 3: Figure S2), and the figures are summarized in a single report (Additional file 5). For the xenograft data the simple T-cell vs. B-cell comparison generated a large number of differentially expressed genes that results in the top GO terms for the genes upregulated in T-cells including “T-cell activation”, “T-cell aggregation” (Fig. 5a). The KEGG analysis top hits include “T-cell receptor signaling pathway” (Fig. 5b). Finally the GSEA has a top hit of “LUPUS_CD4_TCELL_VS_LUPUS_BCELL_DN” and other clearly biologically relevant hits such as “MULLIGHAN_MLL_SIGNATURE_1_UP” (Fig. 5c). The GSEA leading edge enrichment produced by ClusterProfiler for top hits is shown in Fig. 5d.

### Immunology module

**Fig. 6:**
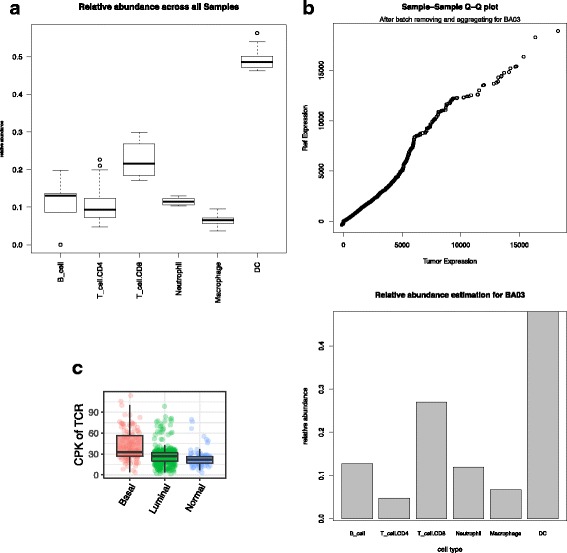
**a** Summary boxplot depicting the population levels of various immune cell classes seen across normal, luminal and basal breast cancers in TCGA. **b** A Q-Q plot that depicts the gene expression of immune cells after batch correction within the TIMER module, and a bar graph per sample that depicts the proportion of immune cell signature in a particular sample. **c** Plots depicting TCR clonal diversity reported as clonotypes per thousand reads (CPK) in normal, luminal and basal breast cancers

While the above functionality is useful to a large fraction of RNA-seq analysis, we illustrate the advantages of the easy extensibility of VIPER with several optional packages, specifically with regards to immunology analysis. VIPER is packaged with the Tumor IMmune Estimation Resource [11] (TIMER), software that estimates the abundance of tumor-infiltrating immune cell types within samples. Given a sample from one of the 23 supported TCGA cancer types set in the config file, a user can perform TIMER analysis that will report the estimated abundance of B cells, CD4 T cells, CD8 T cells, neutrophils, macrophages, and dendritic cells within their samples (Fig. 6a). These immune cell types are linearly separable in the statistical model and represent currently the most promising immunotherapy targets.

In addition to TIMER, VIPER also comes packaged with TRUST, a recently developed method to perform de novo assembly of the hypervariable complementarity-determining region 3 (CDR3) sequences of the T cell receptors from RNA-seq data [10]. For each sample input, after initial alignment, the bam file, including unmapped reads, is used to infer the CDR3 RNA and amino acid sequences based on the contigs assembled from the unaligned reads. Since tumors with higher levels of T cell infiltrates have more TCR reads, resulting in the assembly of more CDR3 sequences, we therefore report the number of unique CDR3 calls in each sample normalized by the total read count in the TCR region, which we visualize in a boxplot as a distribution of clonotypes per thousand (kilo) reads (CPK), as a measure of clonotype diversity (Fig. 6c). The output CDR3 assemblies can be used to study tumor-infiltrating T cells and study the association between the T cell repertoire and tumor somatic mutations, potentially in a correlative manner to predicting tumor neoantigens [10].

### Other conditional analyses

**Fig. 7:**
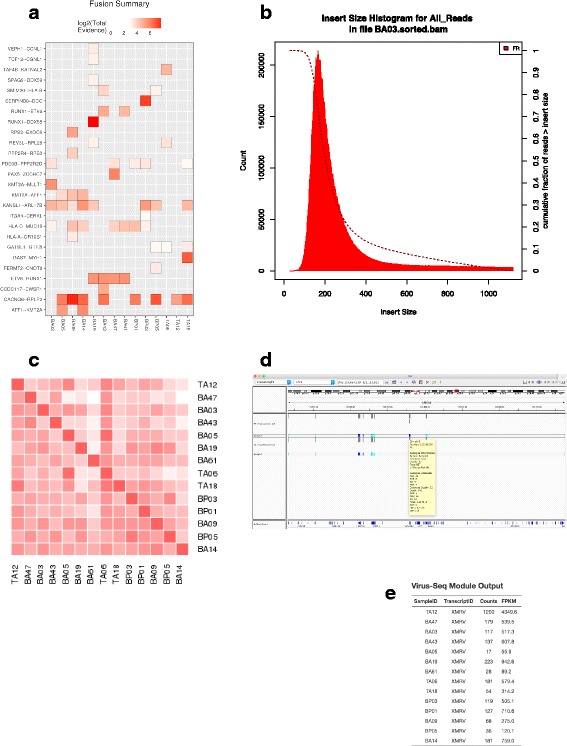
**a** Fusion-Gene Analysis Summary Plot with samples along the x-axis and the fusion genes discovered depicted along the y-axis. **b** Histogram Plot illustrating the insert size per paired end sample. **c** HLA SNP correlation heatmap showing the correlation between the HLA regions of each sample. **d** Example of an IGV snapshot with the full vcf annotation of all SNPs seen genome wide. **e** Table output for the virus-seq module that depicts the top represented viruses within the sample

As mentioned above when the input data are paired end, VIPER uses STAR-Fusion [20, 21] to identify potential fusion genes discovered during alignment. The evidence for the top candidates is put in the report as a heatmap (Fig. 7a). Numerous false positives are seen and so manual curation of the top hits is recommended; in the case of the xenografts all the clinically detected fusions for these samples are also detected in the xenografts [16]. For paired end data the distribution of insert sizes is also generated (Fig. 7b). VIPER also comes packaged with modules that perform whole-genome SNV (single nucleotide variant) calling (human and mouse), viral analysis (human samples only) and batch effect correction which users can enable by toggling flags in the configuration file.

By default, VIPER performs an efficient SNV analysis using the varscan tool [35] on the HLA regions (of the specified species) to help users detect sample swaps/mislabeling events (Fig. 7c). Genome-wide SNV analysis can be enabled using a flag within the configuration file and VIPER will generate results in Variant Call Format (VCF) annotated using SNPeff [36] (Fig. 7d).

VIPER allows users to detect human viral transcripts within their samples. Reads that failed to map during the initial alignment step are re-processed and aligned to a hybrid human assembly that contains a compendium of viral DNA sequences classified as being part of chromosome M [8]. Cufflinks is then used to calculate viral abundance, counts, and FPKM values of the top viral hits. These results are summarized in the VIPER report [37]. For the xenograft samples chosen there were no viruses detected other than a murine virus from the xenograft host (Fig. 7e).

Batch effects are known to be a major problem when combining datasets from different labs or generated with different protocols [38–40]. VIPER incorporates an easily accessible method for implementing batch correction to the analysis using the R library ComBat [41]. VIPER will correct for the batches specified by the user, and output the batch-corrected expression matrix, in addition to the original, and several graphics output by ComBat depicting the correction performed. This batch-corrected matrix is then automatically utilized in all further analysis.

## Discussion

VIPER was designed around a few core concepts that permeate throughout the design of the pipeline. First, VIPER was designed with visualization of results as a key principle with the output encapsulating important analysis results in informative, publication quality figures. Secondly, using Snakemake offers distinct advantages in both efficiency and customizability. Lastly, we wanted to ensure that VIPER could be installed and used by anyone, even those with limited computational experience. Therefore installation of VIPER requires minimal user input and the full pipeline is run using inputs that can be made in any text or table editor and a single terminal command.

### Visualization of data

VIPER outputs a figure or table for all analyses that allows all users to rapidly understand and utilize the analysis results. The most important visualizations are all compiled into a single report file, which highlights the main features of the analysis, while providing explanation of each of the individual processes needed to create the figure. All of the figures are output in pdf or png format, and provide clear explanations of the RNA-sequencing results of the experiment (Additional file 5).

### Snakemake as a framework

VIPER’s Snakemake backbone provides several advantages that set it apart from other sequencing pipelines. VIPER’s “rules” can be composed of tools that are written in a number of languages including R, Perl, Python, *NIX command line tools or even tools written in JAVA or C++. As of Snakemake 3.7 each rule is evaluated in its own environment making it even easier to mix tools (e.g. Python 2.7 and Python 3 based software). This enables VIPER to be flexible in the tools that can be used in the pipeline, permitting construction of a pipeline most appropriate for the data under examination.

Snakemake was built with the concept of parallelization in mind enabling VIPER to make use of its ability to spawn jobs in parallel to maximize its speed and make full use of the provided processing power. For example, RSeQC is the quality control suite that VIPER uses for determination of the quality of the sequencing data. We modified this suite and parallelized the individual tools in addition to adding additional scripts that together enable QC of multiple samples to occur at once, drastically increasing the speed of analysis. This parallelization is also used in many of the steps including the alignment, where the aligner itself is a multi-threaded application, to the downstream analysis, where all of the various differential expression analyses are done in a fashion that maximizes the use of the provided computational power. Additionally, Snakemake has the capability to scale from single-core workstations over multi-core servers to compute clusters of different architectures, without the need to modify the workflow.

Snakemake’s “bottom up” method of determination of job execution allows for a number of advantages including crash recovery and specification of subsetted analyses. A Snakemake workflow is composed of individualized rules, each of which takes a specified input and generates a designated output. Snakemake determines the execution of events by checking timestamps, and as long as a rule was properly executed and the input file timestamps have not changed, then it will not attempt to regenerate the output. If there is a computer or user error during VIPER execution, the output up to the point of failure is not lost, and the user will not need to rerun the whole pipeline.

This feature also enables the user to easily rerun downstream analyses or reprocess subsets of samples without repeating the whole pipeline. VIPER will only execute a rule if its output is required for a later rule, or if its input has been updated. Because of this core concept, subsetting of analyses is as simple as changing the metadata input file. Snakemake will determine via the file timestamps that the aligned data was not changed (just the metadata describing samples) and will proceed to the analysis downstream of alignment (starting with differential expression) thus skipping the computationally intensive upstream rules. With this in mind, we incorporated a simple “analysis token” within the *config* file that enables users to save several different subanalyses while maintaining VIPER’s folder hierarchy (Additional file 3: Figure S2).

Customization of VIPER requires a baseline understanding of the underlying framework of Snakemake, but will allow users to continually update and modify their instance of VIPER. For example, while developing VIPER, we determined that in addition to the SNP scan of the HLA region, we also wanted to build in the option for a genome wide SNP scan. Incorporation of this functionality simply required defining a new “rule” and then adding a flag in the *config* file to turn on the analysis (Additional file 6: Figure S4).

### Ease of use

The methods for installing, deploying, and using VIPER are provided in the Additional file 7, and the documentation is available online. It is worth noting here that VIPER was designed to use the package manager Conda [42] and the Bioconda [43] channel. This allows users to download and install the dozens of tools and packages that go into VIPER with a single command. Setting up a VIPER analysis requires basic usage of the terminal and software such as Excel to edit a comma separated values (csv) file, both of which involve very simple commands.

### Comparison to other tools

VIPER is not the only non-commercial RNA-seq analysis software package available. Other recently published RNA-seq pipelines include HppRNA [12], TRAPLINE [13], and QuickRNASeq [14]. While these pipelines have some features and software packages in common with VIPER, the number of features included, package management software, and reporting functionalities vary considerably (Table 1).

**Table 1:** Comparison of features in VIPER with other RNA-seq pipelines

Features	VIPER	HppRNA	TRAPLINE	QuickRNASeq
Quality Control	X	X	X	X
SNP Detection	X	X	X	X
Fusion Gene Detection	X	X		
Differential Expression	X	X	X	
Pathway Analysis	X		X	X
Consolidated Report	X			X
Galaxy Based			X	
Dependencies Packaged	X	X	X	
Support New Species	X	X		
Package Easy Update	X			X
Batch Correction	X			
Virus Detection	X			
Immunology Analysis	X			

The RNA-seq pipeline HppRNA employs the same Snakemake workflow management platform as VIPER allowing it to share the benefits of this workflow engine. The software offers an impressive number of mapping, quantification and testing algorithms, but this flexibility may be confusing for users primarily interested in data analysis and not benchmarking different alignment algorithms. While it is possible to customize VIPER to use any preferred aligner, the default tools included in VIPER have been curated based on current best practices in the field [4] and a fast runtime. For some analyses, such as differential gene expression testing, it is informative to compare results generated by different algorithms, and indeed this was the motivation for including both Limma and DESeq2 for statistical testing in VIPER. Finally HppRNA does not offer the focus on the visual output we had a main design criterion.

RNA-seq pipelines also display variability in strategies to manage software dependencies. TRAPLINE manages dependences through Galaxy, which provides a helpful user interface, but it requires a Galaxy installation. In contrast, VIPER utilizes the Conda package and environment management system that can be installed and updated easily with only a few commands.

The graphical summary report generated by VIPER is another feature that allows quick and efficient communication and summarization of experimental results. QuickRNAseq also provides a very nice interactive display of the data, but it is not as easily transferable. In contrast, the VIPER report is a self-contained html document that can be attached in an email and opened on a mobile phone.

The other pipelines also have additional features such as SNP detection, gene fusion detection, and pathway analysis, but none except VIPER has all of these features (Table 1). Additionally, the integrated batch-correction, Virus detection and Immunology modules are unique to VIPER. These capabilities are otherwise only available to those who can successfully navigate the installation and implementation of the individual tools. We believe that this represents high value for users requiring such analyses for their samples.

We are currently running the software on both multi-core servers and compute clusters. As not everyone has access to such systems we see an exciting future direction as VIPER being implemented within an Amazon Machine Image that will enable high scalability for anyone. This should be readily achievable with the capabilities of the snakemake framework [15] and make highly scalable analysis more widely available.

## Conclusions

We present a new RNA-seq pipeline VIPER that is fast, efficient, customizable, and easy of use enabling it to be an effective and modern tool for life scientists. We believe that one of VIPER’s most important advantages is that it is a tool built primarily by biologists to run a wide variety of useful analyses, in a manner easy enough to be employed by users without significant computational training. There are new and innovative tools for RNA-seq being created at an extraordinary rate that can further our understanding of the transcriptomic landscape and the easy extensibility of VIPER allows for new approaches to be tested and incorporated as needed. We designed VIPER to incorporate what we believe to be fundamental to gaining a useful understanding of any RNA-seq data set. But it is the authors’ hope that VIPER can be a framework and starting point for others to build upon and further improve VIPER as a tool and ultimately extend our collective ability to extract information from the transcriptome.

## Additional files

Additional file 1: Config Example (YAML 6 kb)

Additional file 2: Metasheet Example (CSV 600 bytes)

Additional file 3: **Figure S2.** (a) Example of the VIPER project folder. The main components are VIPER, DATA, and ANALYSIS with the input files *config.yaml* and *metasheet.csv.* (b) Expanded ANALYSIS folder illustrating the output of VIPER. The plots folder here is expanded to illustrate how the output assumes a simple hierarchical structure, and that each of the clustering figures are associated with a text file containing the underlying information. (PDF 212 kb)

Additional file 4: **Figure S1.** Graphical overview of the computational steps performed by VIPER processing a single fastq file. The nodes of the graph represent the execution of a rule and a directed edge between node A and B means that the rule underlying node B needs the output of node A as an input. A path in the graph represents a sequence of jobs that have to be executed serially, but disjoint paths can be run in parallel. This specific directed acyclic graph (DAG) was automatically generated by VIPER based on the directive to run the rule named ‘target’, using a single fastq file as input. (PDF 436 kb)

Additional file 5: Complete VIPER report in html format (HTML 10310 kb)

Additional file 6: **Figure S4.** (a) Code snippet from the config.yaml file demonstrating the addition of a boolean flag indicating whether or not to run the genome wide SNP scan. (b) Code snippet from the snp.snakefile demonstrating the addition of rules built off of existing output (aligned STAR BAM files) and yielding additional output (genome-wide SNP scans). (PDF 104 kb)

Additional file 7: Implementation and Installation [43–45] (Additional file 8: Figure S3). (DOCX 15 kb)

Additional file 8: **Figure S3.** VIPER was run on a dataset (12 samples; single end data; 36.7 M reads on average) and finished in 24 h. VIPER performance during this run is captured using Ganglia on a 96GB RAM 6 processor Intel Xeon machine. (a) System usage and (b) CPU load captured showing how VIPER is parallelized across 6 processors with (c) ~35G memory utilized for the alignment part of the pipeline. (PDF 79 kb)

## Abbreviations

BAM: Binary alignment/mapping
CDR3: Complementarity-determining region 3
CPK: Clonotypes per thousand (kilo) reads
GO: Gene ontology
PCA: Principal component analysis
SNV: Single nucleotide variant
TIMER: Tumor IMmune Estimation Resource
VCF: Variant call format
VIPER: Visualization Pipeline for RNA-Seq
