# Supplementary material for: VIPER: Visualization Pipeline for RNA-seq, a Snakemake workflow for efficient and complete RNA-seq analysis
Source: BMC Bioinformatics. 2018 Apr 12;19:135. doi: 10.1186/s12859-018-2139-9 (PMC5897949; doi:10.1186/s12859-018-2139-9)
Supplement: Supplementary file 3 — Figure S2. (a) Example of the VIPER project folder. The main components are VIPER, DATA, and ANALYSIS with the input files config.yaml and metasheet.csv. (b) Expanded ANALYSIS folder illustrating the output of VIPER. The plots folder here is expanded to illustrate how the output assumes a simple hierarchical structure, and that each of the clustering figures are associated with a text file containing the underlying information. (PDF 212 kb) [file 12859_2018_2139_MOESM3_ESM.pdf]

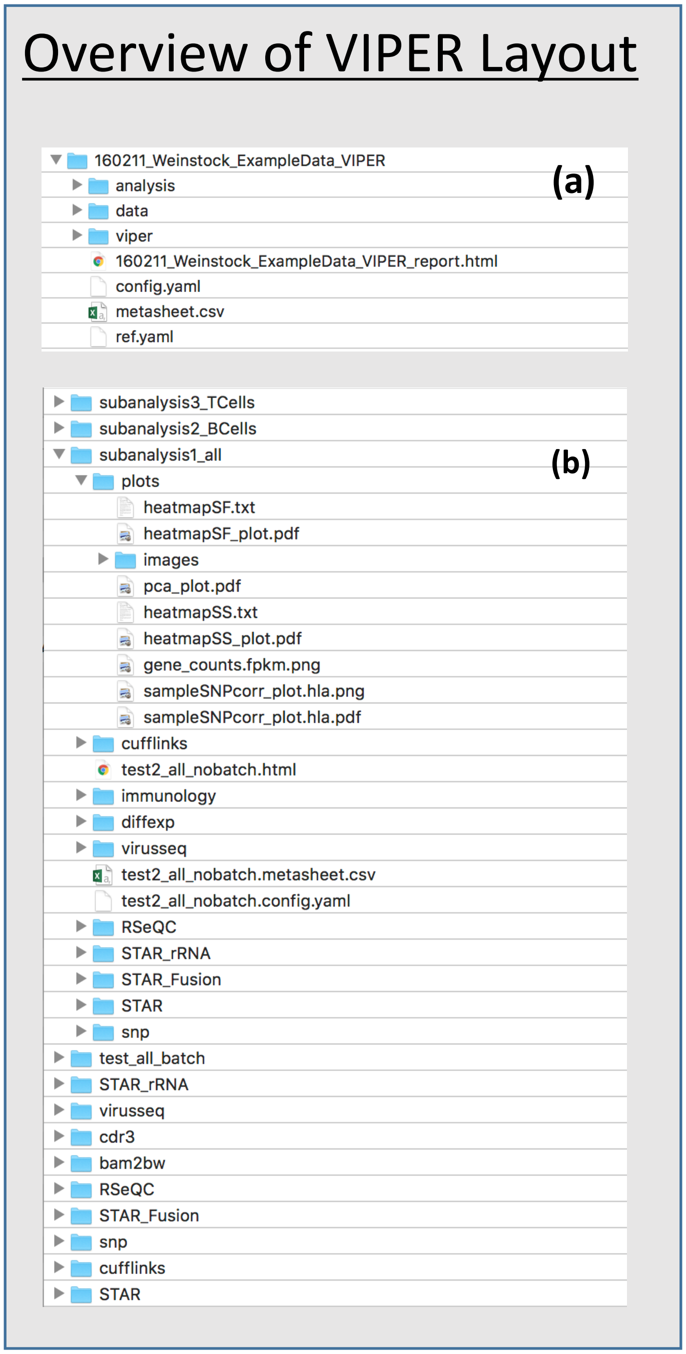

Supplementary Figure 2
