# Supplementary figures and images for: VIPER: Visualization Pipeline for RNA-seq, a Snakemake workflow for efficient and complete RNA-seq analysis
Source: BMC Bioinformatics. 2018 Apr 12;19:135. doi: 10.1186/s12859-018-2139-9 (PMC5897949; doi:10.1186/s12859-018-2139-9)

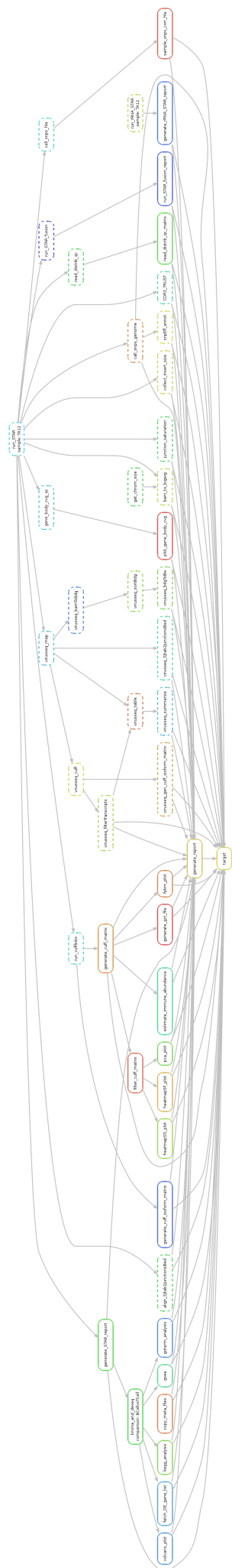

Supplement: Supplementary file 4 — Figure S1. Graphical overview of the computational steps performed by VIPER processing a single fastq file. The nodes of the graph represent the execution of a rule and a directed edge between node A and B means that the rule underlying node B needs the output of node A as an input. A path in the graph represents a sequence of jobs that have to be executed serially, but disjoint paths can be run in parallel. This specific directed acyclic graph (DAG) was automatically generated by VIPER based on the directive to run the rule named ‘target’, using a single fastq file as input. (PDF 436 kb) [file 12859_2018_2139_MOESM4_ESM.pdf]

(a)

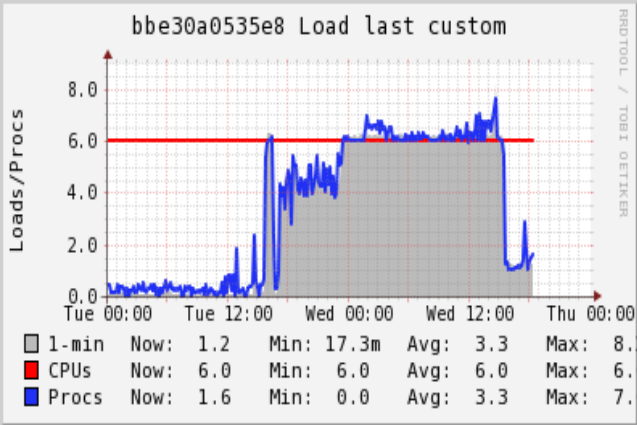

(b)

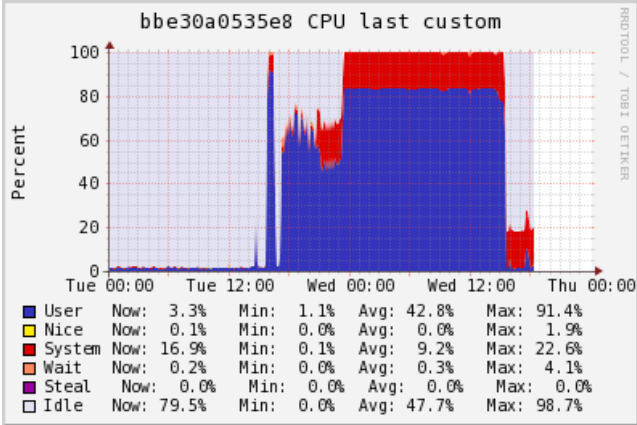

(c)

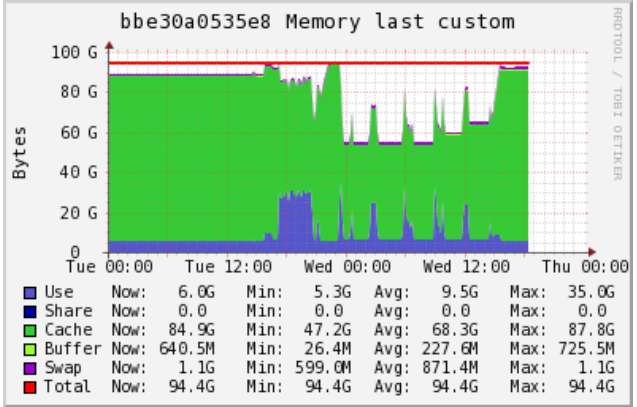

Supplementary Figure 3

Supplement: Supplementary file 8 — Figure S3. VIPER was run on a dataset (12 samples; single end data; 36.7 M reads on average) and finished in 24 h. VIPER performance during this run is captured using Ganglia on a 96GB RAM 6 processor Intel Xeon machine. (a) System usage and (b) CPU load captured showing how VIPER is parallelized across 6 processors with (c) ~35G memory utilized for the alignment part of the pipeline. (PDF 79 kb) [file 12859_2018_2139_MOESM8_ESM.pdf]
