# Supplementary material for: VIPER: Visualization Pipeline for RNA-seq, a Snakemake workflow for efficient and complete RNA-seq analysis
Source: BMC Bioinformatics. 2018 Apr 12;19:135. doi: 10.1186/s12859-018-2139-9 (PMC5897949; doi:10.1186/s12859-018-2139-9)
Supplement: Supplementary file 5 — Complete VIPER report in html format (HTML 10310 kb) [file 12859_2018_2139_MOESM5_ESM.html]

xml version="1.0" encoding="utf-8" ?


VIPER: Visualization Pipeline for RNAseq - len\_original\_run


# VIPER: Visualization Pipeline for RNAseq - len\_original\_run

# Alignment Summary

> Raw reads were mapped or aligned to the reference organism using STAR software.
>
> The **uniquely mapped read counts** and the **total read counts** for all the samples are summarized in the following image. In most cases, more than 70% of the reads should uniquely map to the reference genome.
> Contamination, low quality sequencing data, or poor library contruction may result in <60% uniquely aligned reads.

# Library Prep Quality Metrics

## Read Distribution QC

> This graph displays the disibution of reads mapped to **features** across the genome for each sample. Distribution profiles should be similar across samples.
> A sample with a siginficantly different distribution profile may indicate that it was initially a poor sample or that there was a problem during library preparation.
> **mRNAseq libraries will typically contain less than 20% intronic mapped reads** whereas **totalRNAseq libraries may have greater than 40% intronic mapped reads**.

## rRNA removal QC

> This graph displays the percentage of reads mapping to ribosomal RNA reference sequences. Most RNAseq library prep methods are designed to avoid sampling ribosomal RNAs which typically represent greater than 80% of total RNA. If rRNA removal was effective, less than 5% of the reads should map to rRNA sequences and for mRNA libraries fewer than 1%.

## Genebody Coverage

> For accurate gene expression quantification, mapped reads should be evenly distributed across genebodies.
> Significantly skewed profiles (5' or 3') may introduce quantification bias and/or represent poor quality library preparation.
>
> For example, mRNAseq library preps typically use oligo-dT beads to capture mature transcripts and can be prone to 3' bias in genebody coverage if degraded RNA (RIN < 7) is used as input. This may result in inaccurate gene quantification and the following graphs will help diagnose.
> There are other prep methods that may result in 5' bias too. Ideally, coverage should be uniform across the genebody. The line plots should look like this: "∩"
>
> Figures generated using RSeQC software.
>
> **Line Plot**
>
>
>
> **Heatmap**
>
> This graphic may facilitate identification of biased samples.
>
> Scale: Blue = 0 Pink =1

# Experimental Quality Control

## Principle Component Analysis

> High dimensional expression data are mathmatically reduced to principle
> components that can be used to describe variation across samples in fewer dimensions to allow human interpretation.
> Principle component 1 (PC1) accounts for the most amount of variation across samples, PC2 the second most, and so on. These PC1 vs PC2 plots
> are colored by sample annotation to demontrate how samples cluster together (or not) in reduced dimensional space.
> For more detailed description of Princilple Component Analysis, start with wikipedia.
>
> > This plot indicates how much of the overall variance is described by the principle components in descending order.

## Sample-to-Sample Correlation Heatmap

> This heatmap displays hierarchical clustering of spearman rank correlations across samples.
> Highly correlated samples will cluster together and be represented in red. Samples that do not correlate will be represented in blue.
>
> > Sample-to-Sample data matrix is /analysis/plots/heatmapSS.txt

## Sample-Feature Correlation Heatmap

> This heatmap illustrates hierarchical clustering of samples based on the top 5 percent or roughly 1000 most variable genes or "features."
> The top colomn color annotations are presented to help identify how sample types, groups, or treatments are clustering together (or not).
>
> > What are *these* genes?
> >
> > Data used to generate this sample-feature graphic are in /analysis/plots/heatmapSF.txt

# Fusion Summary

# Differential Gene expression

> Differential gene expression analysis was performed using both limma and DESeq2.
>
> Full analysis output tables are are available in /analysis/diffexp/comparison\_of\_interest
>
> This summary image shows the number of genes that are up regulated and down regulated across each comparison at different adjusted P-value cut-offs.

## Volcano Plots

> Volcano plots are commonly used graphical representations of differentially expressed genes and statistical significance.
> These scatter plots show log2 fold change versus P-value for all genes detected. Each data point represents a gene. Genes are colored red if the log2 fold change is greater than one (log2fc > 1). Genes are colored blue if the log2 fold change is less than negative one (log2fc < -1).
> The plot title indicates the direction of the comparison.
> For example, "treatment\_vs\_control" indicates that genes colored red are up-regulated in the treatment condition compared to the control condition with a statistically significant P-value.

# SNP Plots

## SNP - HLA

# Pathway-Analysis

# Gene-Ontology Annotation

## BCellvsTCell

## MLL.AF4vsTEL.AML1\_B.ALL

# KEGG-Pathway Analysis

## BCellvsTCell


More pathway plots such as, Ribosome\_3,RNA\_transport\_5,Antigen\_processing\_and\_presentation\_4,Osteoclast\_differentiation\_2 - can be found at analysis/len\_original\_run/diffexp/BCellvsTCell/kegg\_pathways/.

## MLL.AF4vsTEL.AML1\_B.ALL


More pathway plots such as, Cell\_adhesion\_molecules\_(CAMs)\_2,Glycolysis\_\_Gluconeogenesis\_4,Histidine\_metabolism\_5,Axon\_guidance\_3 - can be found at analysis/len\_original\_run/diffexp/MLL.AF4vsTEL.AML1\_B.ALL/kegg\_pathways/.

# GSEA

> Gene Set Enrichment Analysis was performed on the significant differentially expressed genes using the clusterProfiler R package

# Virus-Seq Module Output

| SampleID | TranscriptID | Counts | FPKM |
| --- | --- | --- | --- |
| TA12 | XMRV | 970 | 3826.8 |
| BA47 | XMRV | 114 | 431.9 |
| BA03 | XMRV | 117 | 517.3 |
| BA43 | XMRV | 137 | 607.8 |
| BA05 | XMRV | 39 | 172.8 |
| BA19 | XMRV | 279 | 1235.0 |
| BA61 | XMRV | 33 | 94.6 |
| TA06 | XMRV | 141 | 489.9 |
| TA18 | XMRV | 43 | 280.2 |
| BP03 | XMRV | 119 | 505.1 |
| BP01 | XMRV | 95 | 482.2 |
| BA09 | XMRV | 59 | 260.6 |
| BP05 | XMRV | 18 | 90.3 |
| BA14 | XMRV | 181 | 759.0 |

This report is generated using ViPeR version [ d0c40d8 ].

> Copyrights::
> :   mbcf.jpg

Molecular Biology Core Facilities, DFCI | 2018-01-16
