# Supplementary material for: VIPER: Visualization Pipeline for RNA-seq, a Snakemake workflow for efficient and complete RNA-seq analysis
Source: BMC Bioinformatics. 2018 Apr 12;19:135. doi: 10.1186/s12859-018-2139-9 (PMC5897949; doi:10.1186/s12859-018-2139-9)
Supplement: Supplementary file 6 — Figure S4. (a) Code snippet from the config.yaml file demonstrating the addition of a boolean flag indicating whether or not to run the genome wide SNP scan. (b) Code snippet from the snp.snakefile demonstrating the addition of rules built off of existing output (aligned STAR BAM files) and yielding additional output (genome-wide SNP scans). (PDF 104 kb) [file 12859_2018_2139_MOESM6_ESM.pdf]

(a) From config.yaml input file into VIPER

```
#Turn on the following if you want a genome wide snp scan in addition to chr6 scan
# possible value are [true,false]
snp_scan_genome: false
```

(b) From SNP Module of VIPER

```
#------------------------------------------------------------------------------
# snp calling GENOME wide (hidden config.yaml flag- 'snp_scan_genome:True'
#------------------------------------------------------------------------------

rule call_snps_genome:
    input:
        bam="analysis/STAR/{sample}/{sample}.sorted.bam",
        ref_fa=config["ref_fasta"],
    output:
        protected("analysis/snp/{sample}/{sample}.snp.genome.vcf")
    params:
        varscan_path=config["varscan_path"]
    message: "Running varscan for snp analysis genome wide"
    shell:
        "samtools mpileup -f {input.ref_fa} {input.bam} | awk \'$4 != 0\' | "
        "{params.varscan_path} mpileup2snp - --min-coverage 20 --min-reads2 4 --output-vcf > {output}"


rule snpEff_annot:
    input:
        vcf="analysis/snp/{sample}/{sample}.snp.genome.vcf"
    output:
        vcf_annot = protected("analysis/snp/{sample}/{sample}.snpEff.annot.vcf"),
        vcf_stats = protected("analysis/snp/{sample}/{sample}.snpEff_summary.html")
    params:
        snpEff_conf=config["snpEff_conf"],
        snpEff_db=config['snpEff_db']
    message: "Running varscan for snpEff annotation analysis"
    shell:
        "snpEff -Xmx2G -stats {output.vcf_stats} -c {params.snpEff_conf} {params.snpEff_db} {input.vcf} > {output.vcf_annot}"
```

Supplementary Figure 4
