# Supplementary material for: VIPER: Visualization Pipeline for RNA-seq, a Snakemake workflow for efficient and complete RNA-seq analysis
Source: BMC Bioinformatics. 2018 Apr 12;19:135. doi: 10.1186/s12859-018-2139-9 (PMC5897949; doi:10.1186/s12859-018-2139-9)
Supplement: Supplementary file 7 — Implementation and Installation [43–45] (Additional file 8: Figure S3). (DOCX 15 kb) [file 12859_2018_2139_MOESM7_ESM.docx]

**SUPPLEMENTARY TEXT: Implementation and Installation**

VIPER is composed of dozens of Snakemake rules that makes use of tools available for the individual steps in RNA-seq analysis. Downloading and learning to use each of these tools separately would require extensive time and knowledge. To avoid this issue, we developed VIPER in conjunction with the package manager Conda and the Conda channel Bioconda [43]. This enables the user to download all of the tools using a single system, and only requires the installation of Miniconda3, followed by a single command that will install all packages from a provided requirements text file. Each of the tools has specified versions to ensure that they are up-to-date and functional in the context of the pipeline as a whole. In addition, all of this installation can occur within a confined virtual environment, which will not affect the current tools or pipelines installed on the user’s system. The reference files necessary for VIPER are common and available from any normal source such as UCSC. We have also assembled all necessary reference files on an online Dropbox repository (https://www.dropbox.com/sh/ltxbl5gxsqlosg1/AAA0Sx05iJiJVdIL4n5HFox8a?dl=0).

Computationally, due to its Snakemake backbone, VIPER will automatically maximize the computational power available, making it highly effective on all systems. Parallelized threading allows VIPER to complete several jobs at once, while also making use of multi-core processes like the alignment done with STAR effectively and efficiently (**Supp Figure 3**). For example, VIPER was able to run a 12 sample dataset (average of 37 million reads per sample) from start to finish in approximately one day using 96GB RAM and 6 Processors [44].

VIPER can be installed for an individual user, or can be installed in a systemic way that allows for a server to make this tool available for all users. Detailed instruction can be found in the documentation on the open sourceware page of bitbucket [45].

*Input into VIPER:*

The input into VIPER was designed to be as straightforward, and comprehensive as possible. The first part of the input is the config file, which specifies the desired parameters and the list of the locations of the data files. The second part is a metasheet which contains all of the metadata that comprises the design of the experiment and the differential analyses.

The config file specified in YAML format that consists of the parameters necessary for the plotting and analysis further downstream of the pipeline and a list of the paths to the fastq files of the raw data. Although it is recommended that this config be filled out in a simple text editor such as Emacs or Vim, it could also be created using any text editor. A template is provided within the VIPER folder that can be adjusted and edited as desired.

The metasheet contains all of the information about the samples and which comparisons will be used for differential expression analysis. There are three sections to the metasheet. The first section is the first column, which contains the sample names. These sample names match the sample names provided in the configuration file that specify the paths to the location of the data. The second section is the metadata specific to each sample. This could be anything from the cell line, to the conditions and treatment, to a gradient of sizes. This section is completely flexible and is provided by the user; it should represent all relevant experimental/sample characteristics. The last section contains the comparisons. This section is where the user can create columns, each representative of the pairwise difference that the user wants to explore between the subsections of the samples. This allows the user to perform multiple analyses with a single metasheet.

After the project folder and the proper input files are set up, running VIPER requires a single command line entry from within the project folder. VIPER will run the complete pipeline from initial alignment through differential expression analyses. Specific replotting and adjustment of experiment or sample sets is as simple as altering the metasheet. VIPER will detect whenever the metasheet has been altered, and therefore will rerun all of the plotting tools to regenerate images with the new data without having to rerun all of the initial, more computationally intensive sections, such as alignment and quality control. If the user requires a specific image output after making adjustments, the user can specify specific reruns within the command line. A comprehensive list of specific plots is listed within the VIPER documentation.
